# The Effect of Video-Based Education on Activities of Daily Living and Wound Healing of Patients with Total Hip Replacement: Randomised Controlled Trials

**DOI:** 10.3390/nursrep15100356

**Published:** 2025-10-04

**Authors:** Ayse Sinem Tas, Ismet Eser

**Affiliations:** 1 Department of Nursing, Faculty of Health Sciences, Bandirma Onyedi Eylül University, Bandirma, 10200 Balikesir, Turkey; 2 Florence Nightingle Hospital School of Nursing, Demiroğlu Bilim University Şişli, 34381 İstanbul, Turkey; iseser11@gmail.com

**Keywords:** total hip replacement, wound healing, activities of daily living, hip function

## Abstract

**Background and Purpose:** There remains a need for effective and accessible education methods to support recovery after total hip replacement. To evaluate the effects of video-based education on daily living activities and wound healing of patients undergoing total hip replacement surgery. **Methods:** A randomised controlled trial was used. Eligible participants were those aged 18 years and over who had undergone total hip replacement surgery in a training and research hospital. The intervention group received video-based training, while the control group received only routine care. **Results:** Patients in the video-based training group showed significantly greater improvement in daily living activities, hip function, and wound healing on postoperative days 5 and 30 compared to the control group (*p* < 0.01). **Conclusions:** Video-based education significantly improved daily living activities, hip function, and wound healing in patients undergoing total hip replacement. Clinicaltrials ID: NCT06523829

## 1. Introduction

Total hip replacement (THR) surgery is the procedure of removing the femoral head and acetabulum that form the hip joint due to reasons such as osteoarthritis and osteoporosis and replacing them with a prosthesis [1]. THR surgery is one of the most common orthopaedic surgeries performed worldwide. Education and counselling should be provided to patients and their relatives in the preoperative, postoperative and discharge period in order for patients who undergo THR surgery to minimise complications. This will also help them fulfil their daily life activities and to minimise pain. Patient education provided by nurses is an important part of standard nursing care [2,3,4]. The visual and auditory nature of patient education enables it to reach a large number of people in a cost-effective manner without any time constraints [5]. Studies have reported that video-based trainings are simpler and faster assimilated for patients compared to other training methods if they are presented in a comprehensible format. For this reason, video-based trainings are becoming increasingly common in health education [6,7].

Although THR surgery is one of the most common orthopaedic surgeries, significant postoperative complications may develop [8]. Wound infection, haemorrhage and prosthesis dislocation are some of these complications [9]. Patient education provided by nurses is an important part of standard nursing care [2,3,4]. Individualisation of the educational materials to be used for the patient will contribute positively to the increase in the patient’s perception and learning ability and to the acquisition of knowledge and skills [10]. The fact that patient education is visual and auditory allows it to reach a large number of people at an affordable cost without any time constraints. Studies have reported that video-based trainings are simpler and faster assimilated for patients compared to other training methods if presented in an understandable way [6,7]. Despite the growing use of video-based education in clinical settings, there is still a lack of randomised controlled studies evaluating its impact on both daily living activities and wound healing in patients undergoing total hip replacement. Building on this evidence, the aim of this study was to evaluate the effects of video-based education on daily living activities and wound healing in patients undergoing total hip replacement surgery.

## 2. Hypotheses

1. It is expected that the mean scores of the postoperative activities of daily living scale of patients with CAP who received video-based training will be higher than those of patients who did not receive training.
2. It is expected that the mean postoperative Oxford Hip Score scale scores of patients with THR who received video-based training will be lower than those of patients who did not receive training.
3. It is expected that the mean postoperative Patient and Observer Scar Rating Scale scores of patients with CAP who received video-based training will be lower than those of patients who did not receive training.

## 3. Materials and Methods

### 3.1. Design

This single-blinded, parallel-group, randomised controlled trial was conducted in accordance with the CONSORT guidelines for randomised clinical trials. The study was conducted at a single centre and involved video-based education for patients undergoing total hip replacement surgery. The trial was registered at ClinicalTrials.gov (ID: NCT06523829) on 22 July 2024.

### 3.2. Setting and Sample

The study consisted of individuals who were hospitalised for treatment in the Bandırma Training and Research Hospital Orthopaedic Service and underwent total hip replacement surgery. G*Power Version (v3.1.7) programme was used to determine the sample size. In order to find a significant difference between the treatment and control groups, 95% confidence interval, 96% power level, 0.05 error level, 0.14 effect level were calculated and the number of patients required for the groups was determined as 24. Considering the data losses during the research process, 35 patients were included in each group. Among the patients included in the study, the study was terminated with 60 patients in the intervention group due to the death of one patient, one patient’s unwillingness to continue the study, and the inability to communicate with one patient on the 30th day after surgery, and in the control group due to the death of one patient and the inability to communicate with two patients on the 5th day after surgery.

Inclusion criteria

– Those who volunteered to participate in the study;
– Conscious, orientated and co-operative;
– Over 18 years of age;
– Turkish speaking;
– At least primary school graduate;
– Patients who were administered the Standardised Mini Mental Test and scored above 23 points according to the instructions were included.

Exclusion criteria

– People with communication problems (hearing impaired, visually impaired);
– Diagnosed with psychiatric illness;
– Patients who were not allowed to mobilise by the physician.

### 3.3. Randomization and Blinding

A simple randomization method was used to assign patients to the intervention and control groups. The randomization was carried out by an independent statistician who was not involved in the recruitment or implementation phases of the study, in order to minimise selection bias. The allocation sequence was generated using an online randomization tool (https://www.randomizer.org, accessed on 30 April 2022), and a total of 70 patients were randomly assigned to either the intervention or control group in a 1:1 ratio. Each participant had an equal probability of being allocated to either group. The statistician concealed the group assignments until the start of the intervention phase to ensure allocation concealment. Participants were also not informed of their group assignments. However, due to the nature of the study, the researcher who conducted the interventions could not be blinded.

### 3.4. Data Collection Tools

Information form

Consists of 13 questions questioning the patient’s sociodemographic characteristics, who will provide care in the postoperative period, and the status of receiving information about total hip replacement surgery [1,11,12,13].

Standardised mini mental test

It is widely used to assess the cognitive status of elderly individuals. The test can be administered in 10 min and consists of 11 items and five main headings: orientation, recording memory, attention-calculation, recall and language. A maximum score of 30 points can be obtained by giving one point for each correct answer from the SMMT test with a score range of 0–30. As a result of the evaluation of SMMT results, 0–9 points (severe cognitive impairment), 10–19 points (moderate cognitive impairment), 20–23 points (mild cognitive impairment), 24–30 points (normal) are grouped.

Barthel activities of daily living index (Barthel ADL index)

In the study, it was used to evaluate the level of independence of patients in daily activities and Cronbach’s alpha value was calculated as 0.90. The Barthel Index assesses ten domains, including feeding, bathing, dressing, personal hygiene, bowel control, bladder control, toilet use, mobility, stair climbing, and transferring from a wheelchair to a bed. It consists of a total of 30 questions. The total score ranges from 0 to 100, with 0 indicating complete dependence and 100 indicating complete independence. A score of 0–20 reflects total dependence, 21–61 indicates severe dependence, 62–90 represents moderate dependence, 91–99 suggests slight dependence, and a score of 100 indicates full independence.

Oxford hip score (OHS)

It was used to evaluate the hip function of the patients and the Cronbach alpha value was found to be 0.89. The scale used to assess patients’ hip function consists of 12 items that evaluate problems related to the hip joint. These items include the nature of hip pain, ability to go shopping, presence of pain, ability to climb stairs, ability to bathe, walking duration, transportation, night pain, the impact of pain on daily activities, limping, dressing, and rising from a chair. Each item is rated on a 5-point Likert scale, ranging from 1 to 5.

Patient scar assessment scale (PSAS) and observer scar assessment scale (OSAS)

It was used to evaluate the wound site of the patients. Cronbach’s alpha value of the scale was 0.992 for the Patient Scar Evaluation Scale and 0.993 for the Observer Scar Evaluation Scale.

### 3.5. Training Booklet

The training booklet was prepared for participants by the research team using evidence from the literature [14,15,16,17,18,19,20]. The training booklet was given to the patients in the intervention group after they were informed about the subject. Readability and comprehensibility were analysed to evaluate the reliability of the training booklet. Evaluation was made according to the Flesch formula. The prepared training booklet was sent to 5 lecturers who are experts in their fields. Necessary corrections were made in line with the feedback of the experts. The booklet was applied to 5 volunteer patients who underwent THR surgery in the Bandırma Training and Research Hospital Orthopaedics Service. Positive feedback was received from the patients regarding the training booklet. These patients were not included in the study.

### 3.6. Video-Based Training

The content of the video training was derived from the training booklet. The content of the video includes information about the exercises that the patient should do in the early and late postoperative period, the movements that the patient should pay attention to and avoid during movement, how to use auxiliary tools, how to care for the wound site, what are the symptoms of infection that may develop in the wound site, in which cases and how often to apply to the health institution. Professional support was obtained for the filming of the video. The video provides verbal guidance for the prescribed exercises and visually demonstrates their correct execution. The audio instructions are delivered by the researcher, and the exercises are performed by an experienced registered nurse. A warning cross was added to the movements that should not be performed in the video. The total duration of the video is 12 min. The prepared training video was shown to five volunteer patients who underwent THR surgery in the Bandırma Training and Research Hospital Orthopaedics Service and necessary arrangements were made in line with the feedback. The five patients to whom the video was shown were not included in the study.

### 3.7. Data Collection Process

Procedures Applied to Both Groups

During the preoperative period, all participants—both in the intervention and control groups—completed the Individual Introduction Form, the Barthel Index of Activities of Daily Living, and the Oxford Hip Score. All patients received standard nursing care during their hospitalisation. This standard care included patient admission, initial health assessments performed by the nurse on duty, and the implementation of nursing interventions based on the collected data. Information about the surgical procedure and postoperative care was provided by the responsible physician and nurse on duty. Due to rotating shift schedules, different nurses attended to the patients during the preoperative, intraoperative, postoperative, and discharge phases. On the day of surgery, the assigned nurse prepared and transferred the patient to the operating theatre. After surgery, routine monitoring and treatment were carried out in the ward, and discharge instructions were delivered by the nurse on duty.

On postoperative day 5, patients in both groups were evaluated using the Barthel Index of Activities of Daily Living and the Patient and Observer Scar Assessment Scale (POSAS) to assess functional status and wound healing.

On postoperative day 30, follow-up assessments were conducted using the Barthel Index, Oxford Hip Score, and POSAS to evaluate daily living activities, hip joint function, and wound healing. For patients unable to attend the hospital for the 30-day follow-up, data collection was completed via phone interviews conducted by the researcher. To evaluate wound healing remotely, patients were asked to send a photograph of the surgical site. If they were unable to do so due to technical or physical limitations, home visits were arranged. During these visits, the researcher conducted wound evaluations and face-to-face interviews. All evaluations—whether via photograph or home visit—were performed exclusively by the same researcher to ensure consistency across all participants.

Intervention-Specific Procedures

In addition to the procedures described above, patients in the intervention group received a video-based education and a training booklet, in addition to standard nursing care. The educational video, approximately 12 min long, was delivered individually to each patient and their caregiver in the hospital room. This video-based education was provided at three key time points: during the preoperative period, on postoperative days 2–3, and at discharge.

Before hospital discharge, the video was uploaded to the patient’s or caregiver’s mobile device (such as a phone or tablet), enabling them to revisit the material at home as needed.

Patients in the control group, by contrast, received only standard nursing care without any video-based education or additional training materials.

### 3.8. Recruitment Procedures

**Figure 1:**
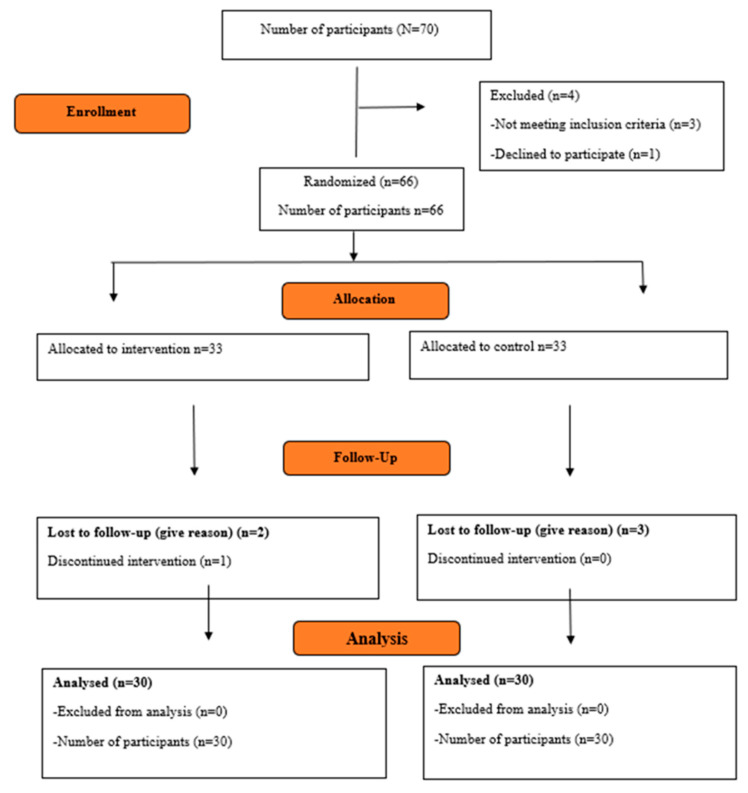
CONSORT flow diagram.

Patients visiting the Orthopaedic Services of Bandırma Onyedi Eylül Training and Research Hospital were screened according to the eligibility criteria. The researcher approached all patients who met the inclusion criteria and invited them to participate in the study. To ensure a high recruitment rate, eligibility criteria and research information materials were provided and explained to staff members of the Orthopedics Services of the participating hospitals in research briefing sessions hosted by the research team. Promotional materials such as posters were also posted around the participating hospitals. See Figure 1 for the study flowchart. The first participation in the research took place on 30 May 2022. The follow-up period of each participant is 30 days. The 30-day follow-up period of the last participant ended on 5 August 2022. Basic data were collected by the researcher. Preoperative and postoperative data of the participants were collected by the researcher. The evaluation made on the 30th day after the surgery was also made by the researcher.

### 3.9. Analysis

IBM SPSS Statistics 23 programme was used for the study data. While evaluating the study data, the suitability of the parameters to normal distribution was evaluated by Kolmogorov–Smirnov test. The suitability of the data for normal distribution was evaluated using the Kolmogorov–Smirnov test. The results indicated that the distribution of the data did not significantly deviate from normality in either the control group (K-S = 0.094, *p* = 0.20) or the intervention group (K-S = 0.087, *p* = 0.28). Since *p* > 0.05 in both groups, the normality assumption was considered satisfied, and parametric tests were applied in subsequent analyses. Descriptive statistical methods (mean, standard deviation, frequency), Student t test in independent groups in the analysis of variables with normal distribution, the relationship between multiple independent variables was evaluated by ANOVA test. Repeated measures analysis of variance was applied in the evaluation of in-group quantitative data, and post hoc pairwise comparisons were performed using the Bonferroni correction. Pearson correlation analysis was used to evaluate the relationship between quantitative variables, Pearson Chi-square and Fisher’s Exact tests were applied to compare qualitative data.

### 3.10. Ethical Considerations

This study was conducted in accordance with the principles of the Declaration of Helsinki. Ethical approval was obtained from the Ege University Medical Research Ethics Committee (Reference Number: E-99166796-050.06.04-404133-829; Decision Number: 21-11T/20). Institutional permission was also granted by Bandırma Training and Research Hospital Orthopaedic Services, where the patients underwent total hip replacement surgery (Date: 16 December 2021, Issue: 44767171-799-E-44767171-799-6065). All participants were informed about the study purpose, procedures, and their rights prior to participation. Written informed consent was obtained from each participant using a standardised information sheet. Participation was entirely voluntary, and individuals were informed that they could withdraw from the study at any time without affecting their medical treatment or relationship with healthcare providers. Potential risks were minimal and primarily related to electronic access to video materials and participation in follow-up phone interviews. However, based on the pilot study, it was unlikely that participants experienced difficulty in using these tools. If any discomfort was reported during the study, participants were provided with appropriate support and counselling independent of the research team and at no cost.

## 4. Results

The findings related to the descriptive characteristics and health status of the patients in the sample are given in Table 1. The mean age of the patients was 71.70 ± 13.73 years, 53.3% were female and 46.7% were male. It was found that 56.7% of the patients would be cared by their children in the postoperative period. It was found that 65% of the patients had chronic diseases and 61.7% of them used medication continuously. The mean scores of Activities of Daily Living before and after surgery (day 5 and day 30) of the intervention and control groups and the comparison between the groups are shown in Table 2. The postoperative (5th day and 30th day) Barthel ADL Index mean scores of the patients in the intervention group were found to be statistically significantly higher than the mean scores of the patients in the control group (*p* < 0.01). In the postoperative period, the intervention group had higher scores in activities of daily living compared to the control group. This indicates that they performed better in carrying out daily living activities than the control group.

The preoperative and postoperative (30th day) Oxford Hip Score Scale mean scores of the intervention and control groups and the comparison between the groups are shown in Table 3. It was found that the postoperative (30th day) mean scores of the patients in the intervention group were statistically significantly lower than the mean scores of the patients in the control group (*p* < 0.01). The fact that the mean OHS was lower in the intervention group after the operation (on the 30th day) indicates that the improvement in hip function was better than in the control group.

The postoperative (5th day and 30th day) Observer Scar Assessment scores of the intervention and control groups and the comparison between the groups are shown in Table 4. It was found that the postoperative (5th day and 30th day) OSA scale mean scores of the patients in the intervention group were statistically significantly lower than the OSA scale mean scores of the patients in the control group (*p* < 0.01). The fact that the mean score of the OSA scale was lower in the intervention group after the operation (on the 30th day) indicates that the wound healing was better than the control group. The postoperative (5th day and 30th day) Patient Scar Assessment score of the intervention and control groups and the comparison between the groups are shown in Table 5. It was found that the postoperative (5th day and 30th day) PSA scale mean scores of the patients in the intervention group were statistically significantly lower than the PSA scale mean scores of the patients in the control group (*p* < 0.01). The fact that the mean score of the PSA scale was lower in the postoperative (30th day) treatment group indicates that the wound healing was better than the control group.

In this study, it was found that the score obtained from the Oxford Hip Score de-creased as the score obtained from the Barthel ADL Index increased. The correlation analysis showed a strong negative correlation between the Barthel Activities of Daily Living Index and the Oxford Hip Score (r = −0.753, *p* < 0.001). As the level of fulfilment of hip joint function increased, the score of the Oxford Hip Score decreased. Patients who can fulfil hip function comfortably are able to provide self-care without any difficulty in performing activities of daily living. Patients score high on the Barthel ADL Index at the level at which they can perform these activities. For this reason, the Oxford Hip Score decreases in patients with increasing Barthel ADL Index scores.

## 5. Discussion

Using educational materials such as video, telemonitoring, web-based trainings that the patient can access after discharge will ensure that the training is permanent and useful [21,22,23]. When the literature was examined, it was found that the post-discharge training by communicating with the patient via WeChat, video training, written document, training booklet and web-based training improved the self-care of the individual and the scores of fulfilling the postoperative IOL increased significantly compared to the preoperative period [21,24,25]. In this study, it was found that the training applied in the evaluation performed on the 5th and 30th postoperative days positively affected the patients’ IOL. Patient education is important in terms of providing joint mobility and fulfilling functional functions. Patient training has been reported to have a positive effect on the patient’s pain, ability to perform joint range of motion and physical function efficiency in the postoperative period [26,27,28,29]. In this study in which video training was performed, the preoperative and 30th day evaluation showed that the hip function of the trained group started to improve and the improvement in hip function continued on the 30th day. Vasantharao et al. [30] reported that wound site problems were encountered in 8.8% of 240 patients who underwent THR surgery. Nurses should observe the wound site after surgery, whether the dressing is wet or not, the presence, colour, amount, amount and type of drainage should be recorded [31,32]. However, information should also be given about wound site infection that may develop. The symptoms of wound site infection and what to do when faced with any of these situations should be explained to the patient. In this study, it was observed that wound healing was better and no infection developed in the wound site evaluation performed on the 5th day and 30th day after surgery in the group that received video training.

In these studies, patient education was provided by using methods such as education booklet, video-assisted education and telephone follow-up. It can be said that video-assisted education is more effective in terms of the educational status of the patients, their age, the fact that visual education is permanent and can be watched repeatedly. In addition, it is thought that such trainings that can be stored in environments such as mobile phones and computers are more advantageous in terms of providing ease of access at any time, not having the risk of loss, and the possibility of patients getting bored while watching these trainings.

## 6. Strength and Limitations

The limitations of this trial are as follows. While video-based education is an effective and accessible method for patient education, facilitating learning through visual materials and enabling better comprehension, certain constraints remain. Accessibility of the educational content even after patient discharge allows for practical application; however, the short-term follow-up period may not sufficiently capture long-term effects. Therefore, longer-term studies are necessary to evaluate sustained impacts on wound healing and activities of daily living. Additionally, the single-centre design of the study may limit the generalizability of the findings to broader populations. Despite these limitations, the trial demonstrates the potential benefits of video-based education in postoperative patient care.

One of these limitations is the variation in patients’ digital literacy levels. Since the intervention primarily relies on video-based education, patients with lower digital literacy may struggle to fully comprehend the content, which could lead to suboptimal outcomes. Additionally, the extent to which patients engage with video education at home may vary based on personal preferences, familiarity with technology, and environmental factors. A more detailed evaluation of these factors could provide valuable insights into how the intervention performs across different patient groups and settings.

## 7. Implications for Practice

It may be recommended to update patient education models and integrate video-based education into patient education programmes. Designing education programmes to encourage patients to actively participate in their own recovery may improve problems that may arise during treatment. Patients are provided with feedback on the education content. Healthcare providers should consider separating video-based education methods from traditional education methods. This may provide more effective results in patient education. Healthcare policies should aim to allocate financial resources to support video-based education and encourage such education programmes. It may be recommended to examine long periods and conduct studies on different patient groups, to compare different educational contents such as animated videos, to use mobile applications and online platforms to increase the accessibility of video-based education, and to develop educational programmes on an individual basis for patients.

## 8. Conclusions

In this study in which video-based patient education was performed, it was found that the patients who were educated in the preoperative and postoperative period were better in fulfilling the ADL and hip joint function in the patient group. It was also found that no wound infection developed. In conclusion, it is recommended that the use of video-based education should be widespread in the education of patients who have undergone THR surgery, the effectiveness of video-based education should be examined in CRediT.

## Figures and Tables

**Table 1:** Distribution of patients according to descriptive characteristics and health status.

Individual Characteristics	Intervention Group (*n* = 30)		Control Group (*n* = 30)		Total (*N* = 60)		Test	
	Mean ± SD		Mean ± SD		Mean ± SD		t	*p*
**Age (year)**	72.10 ± 14.00		71.30 ± 13.69		71.70 ± 13.73		−0.224	0.824
	*n*	%	*n*	%	*N*	%	* χ^2^	*p*
**Gender**								
**Female**	16	53.3	15	50	31	51.7	0.067	0.50
**Male**	14	46.7	15	50	29	48.3		
**After the operation** **carer**							** χ^2^	*p*
**Wife/Husband**	10	33.3	13	43.3	23	38.3		
**Their Children**	17	56.7	17	56.7	34	56.7	0.391	0.183
**Professional Caregiver**	3	10	0	0	3	5		
**Chronic diseases**							* χ^2^	*p*
**Yes**	18	60	21	70	39	65	0.659	0.294
**No**	12	40	9	30	21	35		
**Continuous use of medication**							* χ^2^	*p*
**Yes**	18	60	19	63.3	37	61.7	0.071	0.50
**No**	12	40	11	36.7	23	38.3		

*t*: Independent Sample t Test, * χ^2^ = Pearson Chi-square test, ** χ^2^ = Fisher’s Exact Test.

**Table 2:** Comparison of average daily living activity scores between the intervention and control groups before surgery and after surgery (day 5 and day 30).

	Barthel Activities of Daily Living Index			
	Intervention Group (*n* = 30)	Control Group (*n* = 30)	Test	
	Mean ± SD	Mean ± SD	t	*p*
Before Surgery	86.50 ± 11.45	82.00 ± 18.91	−2.105	0.061
After Surgery Day 5	49.16 ± 9.56	40 ± 12.03	−3.266	0.002
After Surgery Day 30	88.00 ± 14.71	72.83 ± 18.64	−3.497	0.001
Difference between follow-ups	*p*	*p*		
Before Surgery-After Surgery Day 5	<0.001	<0.001		
After Surgery Day 5- After Surgery Day 30	<0.001	<0.001		
Before Surgery-After Surgery Day 30	1.00	0.996		
	F: 80.829	F: 72.975		

**Table 3:** Comparison of average Oxford Hip Score between the intervention and control groups before surgery and after surgery (day 30).

	Oxford Hip Score			
	Intervention Group (*n* = 30)	Control Group (*n* = 30)	Test	
	Ort ± SS	Ort ± SS	t	*p*
Before Surgery	36.7 ± 7.76	37.26 ± 9.17	0.258	0.797
After Surgery (Day 30)	24.73 ± 5.40	31.6 ± 9.07	3.561	0.001
Difference between follow-ups	*p*	*p*		
Before Surgery-After Surgery (Day 30)	<0.001	0.023		
	F: 71.974	F: 5.788		

**Table 4:** Comparison of average Observer Scar Assessment Scale scores between the intervention and control groups after surgery (day 5 and day 30).

	Observer Scar Assessment Scale			
	Intervention Group (*n* = 30)	Control Group (*n* = 30)	Test	
	Ort ± SS	Ort ± SS	t	*p*
OSAS- After Surgery Day 5	28.76 ± 5.91	38.50 ± 11.11	4.235	<0.001
OSAS- After Surgery Day 30	13.6 ± 3.84	23.76 ± 7.84	6.372	<0.001
Difference between follow-ups	*p*	*p*		
OSAS- After Surgery Day 5- OSAS- After Surgery Day 30	<0.001	<0.001		
	F: 632.97	F: 74.59		

**Table 5:** Comparison of average Patient Scar Assessment Scale scores between the intervention and control groups after surgery (day 5 and day 30).

	Patient Scar Rating Scale			
	Intervention Group (*n* = 30)	Control Group (*n* = 30)	Test	
	Ort ± SS	Ort ± SS	t	*p*
PSAS- After Surgery Day 5	28.30 ± 4.28	36.93 ± 7.78	5.321	<0.001
PSAS- After Surgery Day 30	12.63 ± 3.31	23.73 ± 6.63	8.193	<0.001
Difference between follow-ups	*p*	*p*		
PSAS- After Surgery Day 5- PSAS- After Surgery Day 30	<0.001	<0.001		
	F: 925.733	F: 389.88		

## Data Availability

The data supporting the findings of this study are not publicly available in order to protect participant privacy and adhere to ethical standards. However, access to the data may be provided to editors and reviewers upon reasonable request.

## Public Involvement Statement

Patients and caregivers at the hospital were involved in specific stages of this research. In particular, they contributed to evaluating the effectiveness of the prepared educational video in improving patients’ quality of life, determining their level of self-care ability, and preventing wound complications. Additionally, their involvement during the preoperative, intraoperative, and postoperative periods—through ongoing communication, home visits, and participation in the discharge process—provided valuable input to the research process.

## Guidelines and Standards Statement

This manuscript was drafted against the Consolidated Standards of Reporting Trials (CONSORT) guidelines for randomised controlled trial research [33].
